# Both Basal and Acute Restraint Stress-Induced c-Fos Expression Is Influenced by Age in the Extended Amygdala and Brainstem Stress Centers in Male Rats

**DOI:** 10.3389/fnagi.2018.00248

**Published:** 2018-08-22

**Authors:** László Ákos Kovács, Josef Andreas Schiessl, Anna Elisabeth Nafz, Valér Csernus, Balázs Gaszner

**Affiliations:** ^1^Department of Anatomy, Medical School, University of Pécs, Pécs, Hungary; ^2^Center for Neuroscience, Pécs University, Pécs, Hungary

**Keywords:** restraint stress, corticosterone, aging, stress response, amygdala, bed nucleus of stria terminalis, Edinger-Westphal nucleus, dorsal raphe nucleus

## Abstract

The hypothalamus-pituitary-adrenal axis (HPA) is the main regulator of the stress response. The key of the HPA is the parvocellular paraventricular nucleus of the hypothalamus (pPVN) controlled by higher-order limbic stress centers. The reactivity of the HPA axis is considered to be a function of age, but to date, little is known about the background of this age-dependency. Sporadic literature data suggest that the stress sensitivity as assessed by semi-quantitation of the neuronal activity marker c-Fos may also be influenced by age. Here, we aimed at investigating the HPA activity and c-Fos immunoreactivity 2 h after the beginning of a single 60 min acute restraint stress in eight age groups of male Wistar rats. We hypothesized that the function of the HPA axis (i.e., pPVN c-Fos and blood corticosterone (CORT) level), the neuronal activity of nine stress-related limbic areas (i.e., magnocellular PVN (mPVN), medial (MeA), central (CeA), basolateral nuclei of the amygdala, the oval (ovBNST), dorsolateral (dlBNST), dorsomedial (dmBNST), ventral and fusiform (fuBNST) divisions of the bed nucleus of the stria terminalis (BNST)), and two brainstem stress centers such as the centrally projecting Edinger-Westphal nucleus (cpEW) and dorsal raphe nucleus (DR) show age dependency in their c-Fos response. The somatosensory barrel cortex area (S1) was evaluated to test whether the age dependency is specific for stress-centers. Our results indicate that the stress-induced rise in blood CORT titer was lower in young age reflecting relatively low HPA activity. All 12 stress-related brain areas showed c-Fos response that peaked at 2 months of age. The magnitude of c-Fos immunoreactivity correlated negatively with age in seven regions (MeA, CeA, ovBNST, dlBNST, dmBNST, fuBNST and pPVN). Unexpectedly, the CeA, ovBNST and cpEW showed a considerable basal c-Fos expression in 1-month-old rats which decreased with age. The S1 showed a U-shaped age-related dynamics in contrast to the decline observed in stress centers. We conclude that the age- and brain area dependent dynamics in stress-induced neuronal activity pattern may contribute to the age dependance of the stress reactivity. Further studies are in progress to determine the neurochemical identity of neurons showing age-dependent basal and/or stress-induced c-Fos expression.

## Introduction

The first definition of stress was given by Selye (1936) who defined it as a nonspecific response of the body to a potentially threatening demand. The stress machinery is dedicated to maintain the homeostasis and coordinates the adaptive responses (McEwen, 2002; Tsigos and Chrousos, 2002), which may be triggered both by physiological (i.e., hypovolemia, infection) and psychological (i.e., emotional) challenges (Sawchenko et al., 2000; Dayas et al., 2001; Myers et al., 2016).

The key regulator of this adaptive response is the hypothalamus-pituitary-adrenal (HPA) axis (Pacak et al., 1995; Chrousos, 2009; Kino, 2015). The parvocellular part of paraventricular nucleus of the hypothalamus (PVN) releases corticotropin-releasing factor (CRF) inducing adrenocorticotropin (ACTH) excretion from the anterior pituitary (Gragnoli, 2014; Myers et al., 2016) to control glucocorticoid secretion at the adrenal cortex (Myers et al., 2016; Tsigos et al., 2016). Cortisol (in humans) and corticosterone (CORT; in rodents) are dedicated to maintain or restore the homeostasis (Jawahar et al., 2015; Myers et al., 2016). There is no doubt, that the maladaptation of the HPA axis is associated with the development of stress-related mood disorders (Hamon and Blier, 2013; Fischer et al., 2015; Kino, 2015; de Kloet et al., 2016), therefore it is essential, to study how higher order centers contribute to the dysregulation of stress adaptation.

The functional morphological assessment of acute neuronal activity is widely performed by semi-quantitation of immediate early gene (IEG) expression (Kellogg et al., 1998; Chowdhury et al., 2000; Dayas et al., 2001; Kovács, 2008). One of the commonly studied IEG is *c-fos*, which belongs to the Jun/Fos proto-oncogene family. The neural activation may result in depolarization, triggered by various potentially noxious stimuli resulting in transcriptional reprogramming and alteration of cellular phenotype (Senba and Ueyama, 1997; Kovács, 1998, 2008).

Indeed, the acute stress activation of the PVN is widely shown by c-Fos immunocytochemistry (Coveñas et al., 1993; Zhu et al., 2001; Rouwette et al., 2011; Gaszner et al., 2012; de Andrade et al., 2014). Numerous stress-related centers send afferents converging in the PVN (Petrov et al., 1994; Carrasco and Van de Kar, 2003), such as the subdivisions of extended amygdala (Carrasco and Van de Kar, 2003; Ulrich-Lai and Herman, 2009; van-Hover and Li, 2015), the centrally projecting Edinger-Westphal nucleus (cpEW; Otake, 2005) and dorsal raphe nucleus (DR; Van de Kar and Blair, 1999; Ulrich-Lai and Herman, 2009; Lee and Lee, 2014).

Nuclei of the extended amygdala play critical role in stress regulation, anxiety, fear and mood disorders (Fox et al., 2015; Lebow and Chen, 2016). The basolateral (BLA) and medial (MeA) nuclei of amygdala are activated both by acute and chronic stress associated with increased anxiety (for review see McEwen and Gianaros, 2010; Wilson et al., 2015; Ashokan et al., 2016; Lau et al., 2017). The extended amygdala harbors CRF expressing neuron populations (Dabrowska et al., 2013; Nguyen et al., 2016) in the central nucleus of amygdala (CeA), and lateral area of the bed nucleus of the stria terminalis (BNST) to control the HPA axis (Herman et al., 2005; Choi et al., 2007; Callahan et al., 2013). The lesion of the CeA results in decreased anxiety in rodents (Ventura-Silva et al., 2013) and primates (Kalin et al., 2004). The BNST has dual effects on the PVN: the anterior area activates the HPA axis, the posterior part inhibits the HPA response (Choi et al., 2007, 2008). The response of extended amygdala to stressful stimuli is frequently assessed by c-Fos immunolabeling (Kellogg et al., 1998; Chowdhury et al., 2000; Gaszner et al., 2012).

Urocortinergic neurons of the centrally projecting Edinger-Westphal nucleus (cpEW) are associated with changes in activity of the HPA axis (Kozicz, 2007; Gaszner et al., 2009; da Silva et al., 2013; Kormos and Gaszner, 2013) and stress (mal)adaptation in rodents (Gaszner et al., 2004; Neufeld-Cohen et al., 2010; Kozicz et al., 2011; Farkas et al., 2016, 2017; Kormos et al., 2016; Füredi et al., 2017), in non-human primates (Kozicz et al., 2008a) and suicide victims (Kozicz et al., 2008b) as assessed by c-Fos labeling in multiple studies (Gaszner et al., 2004, 2012; Ryabinin and Weitemier, 2006; Okere et al., 2010; Rouwette et al., 2011).

The neurons in the DR are sensitive also to acute stressors (Bouwknecht et al., 2007; Keshavarzy et al., 2015). The serotoninergic neurons in the DR and their involvement in stress, anxiety and affective disorders is well studied as reviewed by Paul and Lowry (2013), Challis and Berton (2015) as well as by Myers et al. (2016).

The above-described centers based on their connectivity to the PVN area may be responsible for the fine-tuning of the HPA axis (Ulrich-Lai and Herman, 2009; Romeo, 2010). This complexity may explain how the wide spectrum of factors (i.e., onset, duration, type of stressor, the gender and the species (Bale and Epperson, 2015; Chaby, 2016; Romeo et al., 2016)) may influence the HPA axis stress response.

Besides these, it is known that the stress response of the HPA axis is also a function of age (Romeo et al., 2006; Koenig et al., 2011) as assessed both by plasma CORT values and by c-Fos expression in the PVN.

Indeed, rats during the first two postnatal weeks show a stress hypo-responsive period (SHRP) characterized by low CORT level and decreased HPA axis sensitivity (Walker et al., 1991; Levine, 1994; Smith et al., 1997). During the SHRP, stress exposure can induce a slight elevation of c-Fos mRNA expression in the PVN, which can be accompanied by elevated CRF mRNA expression without activating the ACTH release and peripheral CORT response. Beyond the SHRP (i.e., from the 20th postnatal day on) stress induces much greater c-Fos expression in PVN associated with HPA axis activation (Smith et al., 1997; Dent et al., 2000).

Pre-pubertal rats also show accelerated c-Fos recruitment in the PVN after restraint compared to young adults when samples were taken 30 min after beginning of the restraint stress exposure (Romeo et al., 2006). In contrast, the comparison of adult and pre-pubertal rats revealed that there is no difference in c-Fos expression in the PVN 45 min after the termination of the acute stress exposure (Romeo et al., 2006). Pre-pubertal animals display also prolonged CORT response to stress compared to the adults (Romeo et al., 2006; McCormick et al., 2010). However, the 21–23 months old Lewis rats show increased sensibility and c-Fos expression in comparison to young adult animals in the PVN (Meyza et al., 2007). Basal CORT levels in old rats were shown to be elevated compared to young adults and middle-aged adults in various species (Lupien et al., 2005; Koenig et al., 2011).

Only few studies compared the c-Fos reactivity of the stress-responsive brain areas in various age groups leading to somewhat inconsistent results probably due to strain differences. Romeo et al. (2006) found more promptly activated c-Fos cells in the pre-pubertal rat PVN vs. young adults. However according to Kellogg et al. (1998) a broader spectrum of neurons and centers were activated in the young adults than pre-pubertal Long–Evans rats. In contrast, Viau et al. (2005) using Sprague Dawley rats detected decreased c-Fos expression in the PVN upon 30 min restraint exposure. Finally, aged Lewis rats (21–23 months old) exert decreased neuronal responsiveness in the MeA, CeA and hippocampus in contrast to young adults, while the neuronal activity in the PVN was found to be elevated (Meyza et al., 2007).

Based on these, the idea arises that the c-Fos stress reactivity of brain areas involved in the regulation of the HPA axis may also be a function of age. To the best of our knowledge, no study was published until now performing a throughout-lifespan systematic comparison of c-Fos immunoreactivity in the above introduced stress sensitive centers of the rat brain. Therefore, we aimed to semi-quantify c-Fos immunosignal and HPA axis activity in control and acute restraint stress exposed rats in eight age groups (i.e., 1, 1.5, 2, 3, 6, 12, 18 and 24 months of age). The hypothesis of this study was that the age-related changes in the HPA axis reactivity to acute restraint stress may be underlined by age-dependent dynamic changes of c-Fos expression in the parvo-and magnocellular divisions of the PVN, in the nuclei of the extended amygdala (MeA, CeA, BLA, ovBNST, dlBNST, dmBNST, vBNST, fuBNST), in the cpEW and DR. In order to determine whether the hypothetic age-related dynamics of c-Fos expression is specific for these stress-related centers, the primary somatosensory barrel cortex (S1) field has also been selected for quantitation.

## Materials and Methods

### Animals

Seventy-three albino male Wistar-R Amsterdam rats bred in the animal facility of the Department of Anatomy (University of Pécs) were used. Studies were performed in eight age groups according to Table 1. Animals were housed in standard polycarbonate cages (40 × 25 × 20 cm) on neutral temperature (24°C) in humidity controlled environment. Rats had free access to standard rodent chow and tap water *ad libitum*. Rats were housed in 2–3 animals per cage groups on 12 h light/dark cycles with light phase starting at 6:00 am. Regular cage cleaning was performed twice a week. Rats were weighed once a week and at the time of final regular cage cleaning. The studies were approved by the Ethics Committee on Animal Research of Pécs University (license No: BA02/2000-25/2011) based on the European Communities Council Directive of 24 November 1986 and the Law of 1998, XXCIII, on Animal Care and Use in Hungary. All efforts were made to minimize the number of animals used and their suffering.

**Table 1:** Experimental design, bodyweight data and number (N) of animals per group.

Groups			
Age	Stress level	Bodyweight (g)	N
1 month	Control	77.4 ± 4.49	5
	Restraint	76.8 ± 4.38	4
1.5 months	Control	152.2 ± 5.60	5
	Restraint	153.6 ± 2.89	5
2 months	Control	262.6 ± 7.78	5
	Restraint	263.6 ± 5.97	5
3 months	Control	381.2 ± 7.12	5
	Restraint	389.5 ± 10.08	5
6 months	Control	407.2 ± 15.88	5
	Restraint	402.6 ± 7.67	5
12 months	Control	527.5 ± 16.21	4
	Restraint	523.75 ± 11.04	4
18 months	Control	547.0 ± 10.90	4
	Restraint	547.75 ± 13.30	4
24 months	Control	510.75 ± 10.88	4
	Restraint	508.5 ± 8.92	4

*Stressed rats were exposed to 60 min restraint stress, while control animals remained undisturbed in their cages until perfusion. Bodyweight data reflect the average of the group ± standard error of the mean (SEM) expressed in grams (g)*.

To minimize possible error due to variation in the sample’s storage time and considering capacity limitations in the animal facility, the breading procedure was planned in a way that the rats reached the required age within a 4 weeks period of time: in the first step the 2, 3, 6 and 24-month-old rats were killed. One month later, the 1, 1.5, 12 and 18-month-old rats were euthanized.

### Acute Stress Protocol

Half of rats in each age-group were exposed to a 60 min restraint stress between 8 am and 9 am. Animals were closed into custom-made conical polycarbonate restrainer tubes with several ventilation holes. The restrainer tube behind the inserted animal was closed with a plug made from cotton wool secured with adhesive tape. For 1-month-old rats a 30 mm, for 1.5-month-old rats a 35 mm, for 2-month-old rats 40 mm diameter perforated polycarbonate restrainer tubes were used. Subjects of all other groups were closed into plastic tubes with 45 mm diameter and 200 mm length. In case of the 12, 18 and 24-month-old rats, the tube was closed, but no cotton wool plug was used for place restriction. The ideal restrainer tube diameter for each age group was determined based on preliminary tests: for each group that largest diameter was preferred, which was still narrow enough to prevent that the animal turned around in the restrainer. After restraint, rats were returned to their original home cages for 60 mins. Control rats of all age groups were left undisturbed in their home cages.

### Tissue Collection and Sample Preparation

Sixty minutes after the end of restraint stress exposure animals were deeply anesthetized by an overdose of intraperitoneal urethane (2.4 g/kg) injection. To avoid the potential acute effect of stress caused by handling and anesthetic injection on CORT levels, the injections for all rats in the same cage were given by two colleagues simultaneously within a time period of 1 min. Only those rats were used in this experiment, which got unconscious within 2 min after injection.

After opening of the chest cavity, a small cut was made on the left ventricle. Blood samples (1.5 ml) were collected into ice chilled plastic tubes pre-filled with 150 μl 7.5 m/m% ethylene-diamine tetra acetic acid. Then, rats were transcardially perfused with 50 ml 0.1 M phosphate buffered saline (pH = 7.4) followed by 250 ml ice cold 4% formaldehyde solution in 0.2 M Millonig sodium-phosphate buffer (pH = 7.4) in 20 min. Subsequently, animals were decapitated and their brains were dissected and post-fixed in the same fixative.

All the brains were sectioned within 2 weeks after the perfusion. Thirty micrometer coronal sections were cut between the optic chiasm and ponto-medullary transition using Leica Vibratome (Leica Biosystems, Wetzlar, Germany). Three series of sections each interspaced by 90 μm were collected into anti-freeze solution (30% glycerol, 20% ethylene-glycol, 0.1 M PBS) and stored on −20°C till further examination.

### Corticosterone Radioimmunoassay

The blood samples were centrifuged on 3,500 rpm for 5 min. Fifty microliter plasma aliquots were stored at −20°C until radioimmunoassay (RIA). To avoid that the circadian CORT rhythm increases the error of our results, blood samples were collected in the same period of time between 8 am and 10 am.

The RIA was performed exactly as published earlier (Gaszner et al., 2004, 2009). Briefly, a mixture of 5 μl of plasma was extracted. The dried extract was reconstituted with assay buffer from which two parallel determinations were made. Each tube contained 500 μl extract, tritiated corticosterone (1,2000 cpm; NET-399, 90-120 Ci/mmol Perkin Elmer, Akron, OH, USA) and 15 nl/tube CS-RCS-57 antibody (1:4,7000 final dilution, Jozsa et al., 2005) in total volume of 700 μl. For standard, Calbiochem CORT was used. After an overnight incubation at 4°C, the bound and free steroids were separated with dextran-coated charcoal. The radioactivity was measured in a two-phase liquid scintillation system. The sensitivity of the assay is 30 fmol/tube. The inter and intra-assay coefficients for variation were 9.13% and 6.5%, respectively.

### Free Floating Diaminobenzidine Immunohistochemistry for c-Fos

The c-Fos labeling was carried out in two steps, as the free-floating technique with 73 vials was not manageable in one run. We randomized our samples in a way that from each experimental group half of the animals were selected for staining in the first run. Then, in a second step, we continued with the labeling of the remaining samples. The two runs were performed in the same week. All the reagents used were from the same vials of the products/kits detailed below. All efforts were done to keep all the considerable conditions constant between the two runs.

Nevertheless, to prove that the two separate staining runs did not influence our results, two approaches were used. First, as an internal control, we included additional series of six stressed rats of different age groups into the second run also which were already processed in the first run. Then, cell counts were compared between the first and second run within the same animals by *t*-tests. Here we confirmed, that there was no difference detectable between the first and second step. Second, we also tested if the two steps influenced our results by including this into the analysis of variance (ANOVA) as an additional factor. The test did not find the main effect of the run significant; therefore, the corresponding results obtained in the two runs were fused and analyzed together.

Besides these above-described technical modifications, the c-Fos labeling procedure was performed exactly as published earlier (Gaszner et al., 2009, 2012). Briefly, sections were washed for 6× 10 min in 0.1 M PBS, permeabilized with 0.5% Triton X-100 (Sigma Chemical). Subsequently, after an incubation in 2% normal goat serum (NGS, Jackson Immunoresearch Europe Ltd., UK) in PBS for 30 min, sections were treated in polyclonal rabbit c-Fos antiserum diluted to 1:500 (Santa Cruz Biotechnology Inc., sc-52, Santa Cruz, CA, USA) in PBS for 16 h at room temperature. After PBS washes, sections were incubated in biotinylated goat anti-rabbit IgG diluted to 1:200 in PBS and 2% NGS (Vectastain ABC Elite kit, Vector Laboratories, Burlingame, CA, USA). After PBS washes, preparations were treated with avidine-biotin complex solution in PBS (Vectastain ABC Elite kit). After PBS rinses, the immunolabeling was visualized in Tris-buffer containing 0.02% diaminobenzidine (D5637; Sigma Chemical, Zwijndrecht, Netherlands) and 0.03% H_2_O_2_. The reaction was controlled under stereomicroscope and stopped by PBS after 7 min. The sections were mounted to gelatin slides, cleared with xylene, air dried and covered slipped with DePex (Fluka, Heidelberg, Germany).

The specificity and sensitivity of our c-Fos antiserum (Sc-52, Santa Cruz) was tested earlier in the rat (Gaszner et al., 2004, 2009). Omission of the primary or secondary antisera, their replacement with normal non-immune sera abolished the immunosignal in this experiment also. Preincubation of the working dilution of the antiserum with the respective blocking peptide (sc-52P, Santa Cruz) prevented the immunolabeling also (images not shown). Western blot analysis supports the specificity of the serum, as published on the homepage of the supplier.

### Microscopy and Digitalization

Sections were selected based on the comparison with the images in the Paxinos and Watson (2007) rat brain atlas. The following brain areas were selected (the numbers in brackets represent the distance of the selected coronal planes from Bregma): CeA [(−2.40 mm)–(−2.92 mm)], BLA [(−2.16 mm)–(−2.92 mm)]), MeA [(−2.52 mm)–(−3.24 mm)]), parvo-(pPVN) and magnocellular (mPVN) divisions of the PVN [(−1.56 mm)–(−1.92 mm)]), cpEW [(−5.16 mm)–(−6.72 mm)]), DR [(−6.84 mm)–(−7.68 mm)]). BNST sections were collected at the planes between +0.12 mm to (−0.24 mm) to the Bregma and the following areas were studied: ovBNST, dmBNST, dlBNST, vBNST and fuBNST. The BNST sub-regions based on the parcellation of Dong et al. (2001) were assessed (see also Hammack et al., 2010). To test whether the expected changes were restricted to the above listed stress-related brain areas, the primary somatosensory (barrel) cortex area [between −3.12 mm–(−3.48 mm) to the Bregma] was also assessed. Here we decided to count the neurons in a 500 × 300 μm large area framing a lamina IV barrel, based on the work by Bisler et al. (2002).

An experienced neurohistologist colleague who was unaware of the identity of the preparations digitalized the sections by Nikon Microphot FXA microscope with a RT camera (Nikon, Tokyo, Japan). The cell counts were determined by simple manual cell counting on five non-edited digital photos on the entire cross-section surface area of each nucleus. The same person for each nucleus performed the cell counting on all images to minimize human bias. A second person supervised the cell counts on randomly selected images, and only confirmed data were used in the statistical assessment. To avoid the error caused by the difference in the cross section surface areas of slightly different cut planes of the same nucleus, the average for each nucleus was calculated based on the five sections. This value represented the cell count of one brain region for one animal. As all groups consisted of 4–5 rats, 4–5 cell count data represented the group in the statistical evaluation.

For publication purposes, selected representative digital images were grayscaled, contrasted, cropped and edited into image montages (Figures 2–7) using Adobe Photoshop 7.0.1 software.

**Figure 1:**
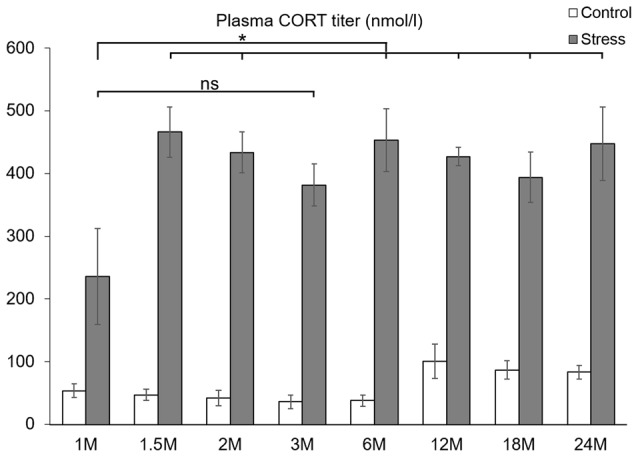
Comparison of plasma corticosterone (CORT) values (nmol/l) of eight age groups. Open bars represent control groups; gray columns refer to stress-exposed rats (*n* = 4–5). As stress induced a significant CORT increase in all age groups (*p* < 0.005) these statistically significant differences were not marked by asterisks. *Post hoc* tests revealed that stress induced CORT response in the youngest rats was significantly lower than in all older ages (*p < 0.05, according to Tukey’s *post hoc* test) except for the comparison with 3 months (M) old rats (ns, not significant *p* = 0.09).

**Figure 2:**
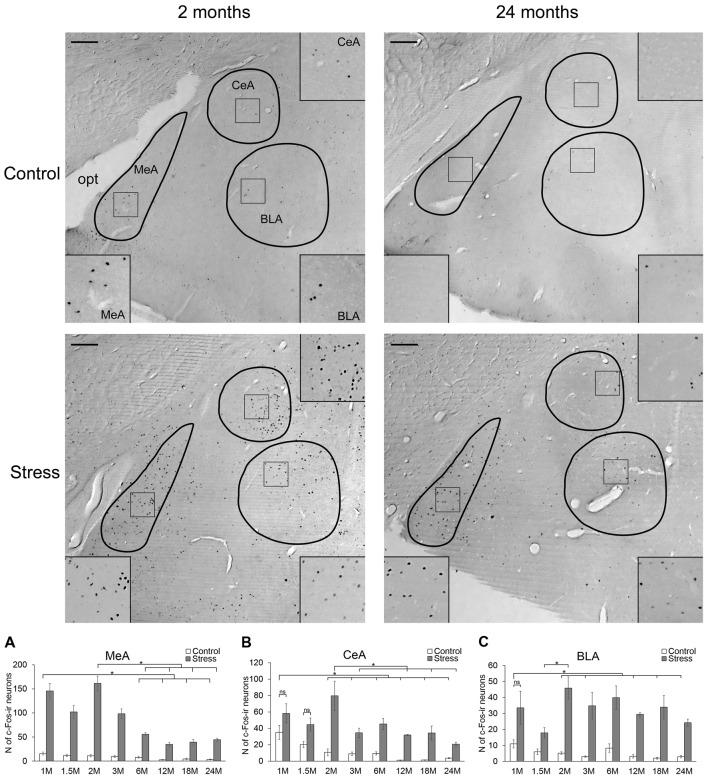
Age-dependent c-Fos expression in the amygdala. Representative images of 2- and 24-month (M) old control and acute restraint stress exposed rats. Insets show higher magnification images marked by boxes in the respective central (CeA, right top corner), basolateral (BLA, right bottom corner) and medial (MeA, left bottom corner) divisions of the amygdala. The number of c-Fos immunoreactive nuclei was compared among eight age groups as demonstrated in panel **(A)** for MeA, in **(B)** for CeA and in **(C)** for BLA. All three examined divisions of the amygdala reacted with a significant c-Fos rise to acute restraint exposure, except for the CeA and BLA of 1 M old rats and the CeA of 1.5 M old animals (ns, not significant). (Due to the lack of significant interaction in the two-way analysis of variance (ANOVA) between age and stress the stress-related c-Fos rise was confirmed by Student’s *t*-tests in the MeA. See also in Table 5). Open bars represent the control groups; gray columns refer to stress exposed rats (*n* = 4–5). opt: optic, tract. *p < 0.05, according to Tukey’s *post hoc* test. Scale bars: 100 μm.

**Figure 3:**
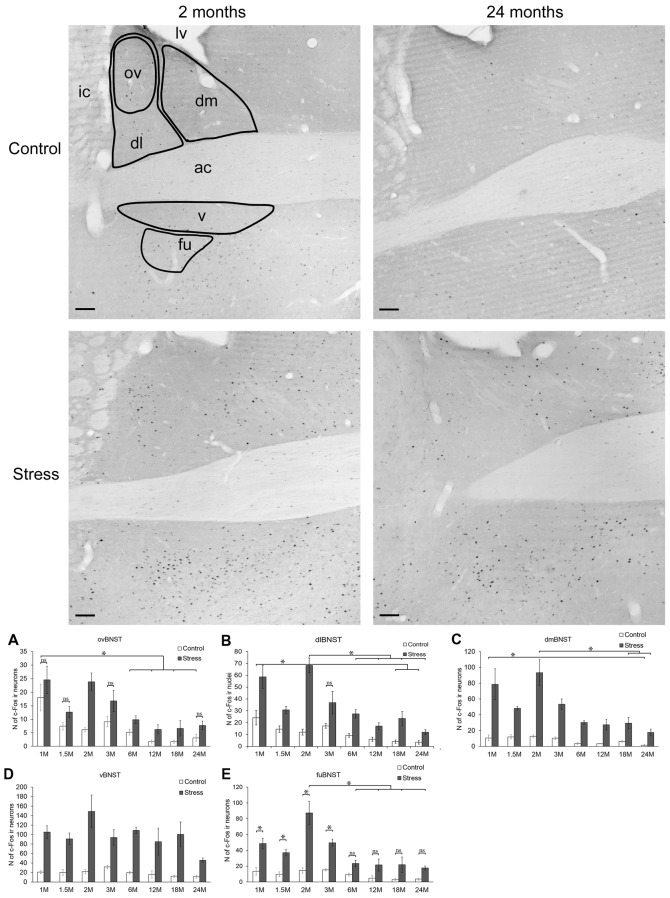
Age-dependent c-Fos expression in the bed nucleus of the stria terminalis (BNST). Representative BNST images of 2- and 24-month (M) old control and acute restraint stress exposed rats. The number of c-Fos immunoreactive nuclei was compared among eight age groups in histograms as demonstrated in **(A)** for the oval (ov), in **(B)** for dorsolateral (dl), in **(C)** for dorsomedial (dm), in **(D)** for ventral (v) and in **(E)** for the fusiform (fu) subdivisions of the BNST. As in dmBNST and vBNST, stress resulted in a significant c-Fos rise in all age groups this fact was not highlighted by asterisks. Open bars represent the control groups; gray columns refer to stress exposed rats (*n* = 4–5). ac, anterior commissure; ic, internal capsule; lv, lateral ventricle; *p < 0.05, according to Tukey’s *post hoc* test, The Student’s *t*-test was used for the ovBNST, dlBNST and vBNST for control vs. stress comparisons and only the not significant (ns) differences were marked (See also in Table 5). Scale bars: 100 μm.

**Figure 4:**
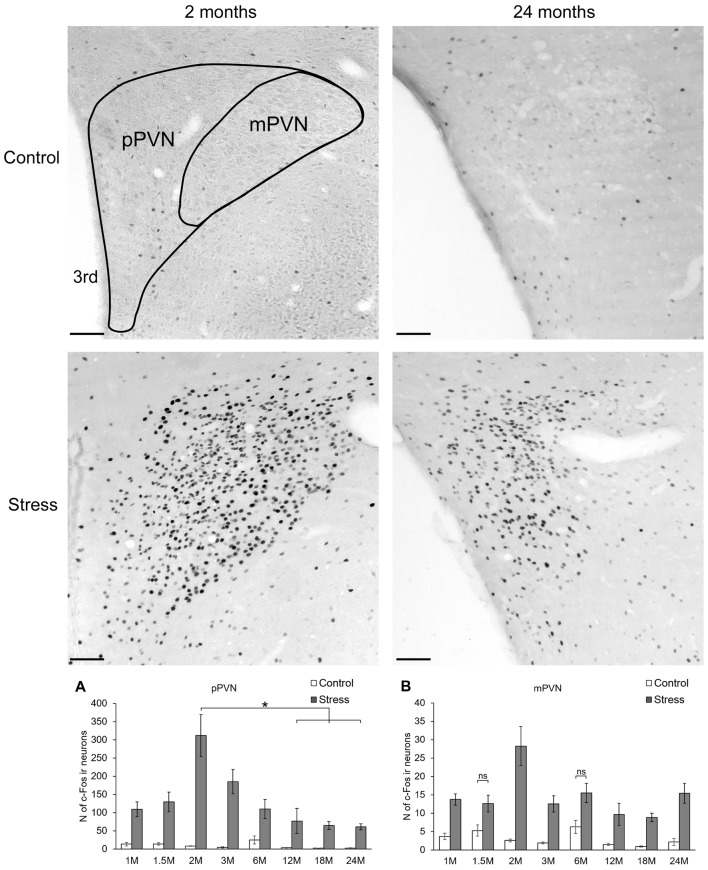
Age-dependent c-Fos expression in the parvo-(pPVN) and magnocellular division of the paraventricular nucleus of the hypothalamus (mPVN). Representative images of 2 and 24-month (M) old control and acute restraint stress exposed rats. The number of c-Fos immunoreactive nuclei was compared among eight age groups in histogram **(A)** for pPVN and **(B)** for the mPVN. As stress resulted in a significant c-Fos rise in all age groups in the pPVN, this fact was not highlighted by asterisks. Open bars represent the control groups; gray columns refer to stress exposed rats (*n* = 4–5). *p < 0.05, according to Tukey’s *post hoc* test 3rd, third ventricle, ns, not significant. Scale bar: 100 μm.

**Figure 5:**
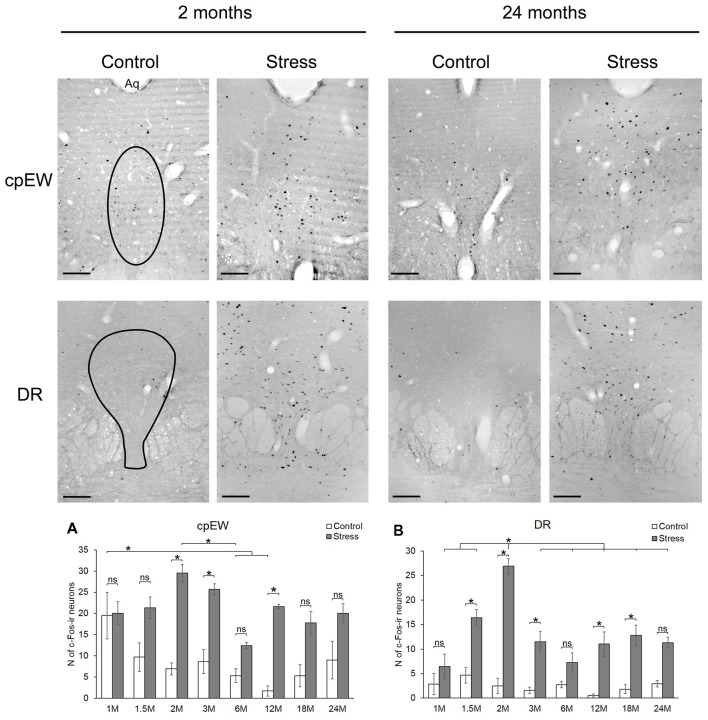
Age-dependent c-Fos expression in the centrally projecting Edinger-Westphal (cpEW) and dorsal raphe (DR) nuclei. Representative images of 2- and 24-month (M) old control and acute restraint stress exposed rats. The number of c-Fos immunoreactive nuclei was compared among eight age groups in histogram **(A)** for cpEW and **(B)** for the DR. Open bars represent the control groups; gray columns refer to stress exposed rats (*n* = 4–5). Aq, cerebral aqueduct; ns, not significant. *p < 0.05 according to the Tukey’s *post hoc* test. Scale bar: 100 μm.

**Figure 6:**
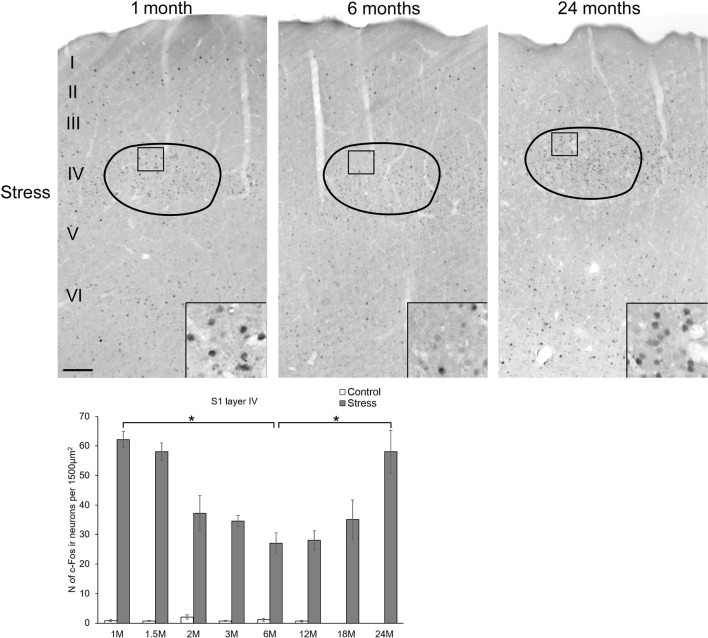
Age-dependent c-Fos expression in the somatosensory barrel cortex (S1). Representative images of 1, 6 and 24-month (M) old acute restraint stress exposed rats. Boxed areas are shown in higher magnification insets in the right bottom corner of the respective low magnification image. Roman numbers represent the cortical layers starting with the pial surface on top. The number of c-Fos immunoreactive nuclei in one lamina IV barrel was compared among eight age groups in histogram **(A)**. Open bars represent the control groups; gray columns refer to stress exposed rats (*n* = 4–5). *p < 0.05 according to the Tukey’s *post hoc* test. The stress effect was confirmed by Students’ *t*-tests in all age groups as no significant interaction was found between age and stress according to the two-way ANOVA. (See also Table 5). Scale bar: 100 μm.

**Figure 7:**
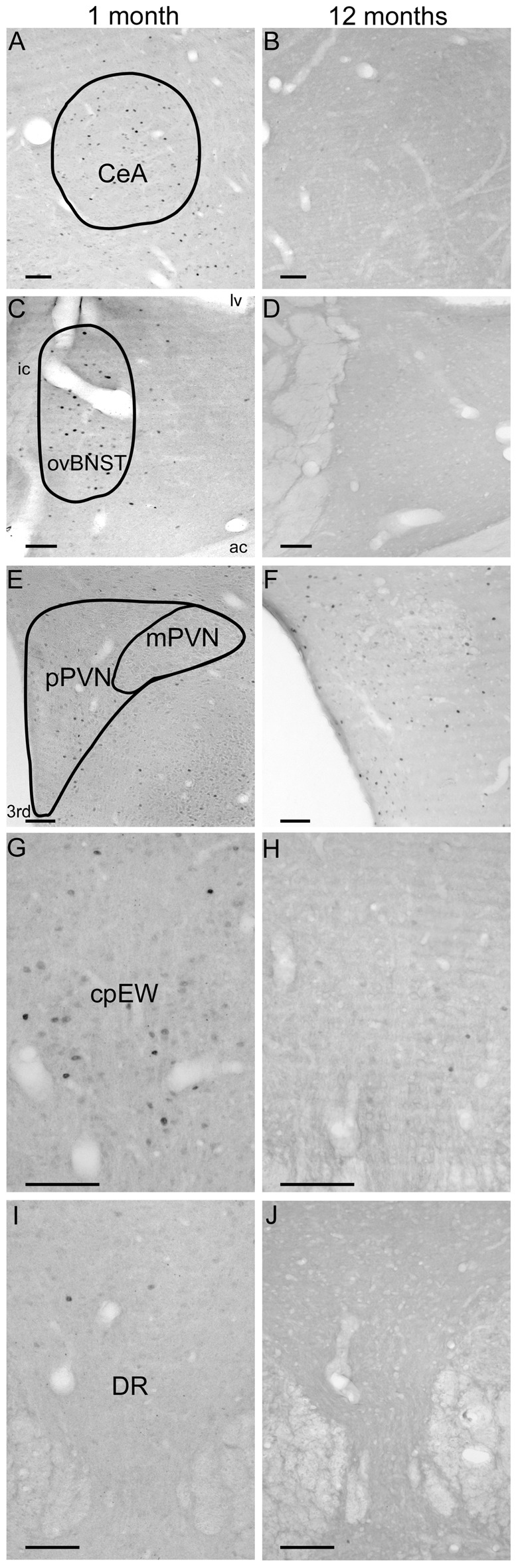
Age-dependent basal c-Fos expression in the central nucleus of amygala (CeA, **A,B**), oval division of the bed nucleus of the stria terminalis (ovBNST, **C,D**), parvo- (pPVN) and magnocellular division (mPVN) of the paraventricular nucleus of the hypothalamus (**E,F**), centrally projecting Edinger-Westphal nucleus (cpEW, **G,H**) and dorsal raphe nucleus (DR; **I,J**). Panels **(A,C,E,G,I)** represent control rats at 1 month of age. Images **(B,D,F,H,J)** show unstressed animals aged 12 months. Note the relatively considerable basal c-Fos expression in the CeA, ovBNST and cpEW in 1-month-old rats. In contrast, c-Fos was nearly undetectable in the PVN and DR in both age groups. ac, anterior comissure; ic, internal capsule; lv, lateral ventricle, 3rd third vertricle. Scale bars: 100 μm.

### Statistical Analysis

All data were presented as mean of groups ± standard error of the mean (SEM). The normality of data distribution and the homogeneity of variance were verified by Shapiro and Wilk (1965) and Hartley’s chi-square tests (Snedecor and Cochran, 1989), respectively. A square root mathematical transformation was applied to obtain normal data distribution in case of CORT values. For the same purpose, cell count data were subjected to logarithmic transformation. Statistical analyses were performed by two-way ANOVA followed by Tukey’s *post hoc* tests. Two-way ANOVA did not find significant interaction of age and stress in six areas (see Table 2). In order to show the age dependency of c-Fos expression, these data were further analyzed by one-way ANOVA for the control and stress condition, respectively. The comparison of pairs of groups was performed by the Tukey’s *post hoc* test using Statistica 8.0. software (StatSoft, Tulsa OK, USA). However, two-way ANOVA found the main effect of stress highly significant in all these five brain areas, the difference between the pairs of groups at all age was verified by Student’s *t*-test also (see Table 5).

**Table 2:** Summary of results obtained in two-way analysis of variance (ANOVA) on c-Fos cell counts.

Area	Two-way ANOVA					
	Main effects				Stress × age	
	Stress		Age		interaction	
	*F*_(1,57)_	*p*	*F*_(7,57)_	*p*	*F*_(7,57)_	*p*
MeA	**517.11**	**<10^−6^**	**16.05**	**<10^−6^**	0.73	0.65
CeA	**234.27**	**<10^−6^**	**17.75**	**<10^−6^**	**8.94**	**<10^−6^**
BLA	**252.76**	**<10^−6^**	**3.45**	**<0.005**	**3.02**	**<0.01**
ovBNST	**51.24**	**<10^−6^**	**13.61**	**<10^−6^**	1.24	0.29
dlBNST	**47.43**	**<10^−6^**	**15.110**	**<10^−6^**	2.00	0.07
dmBNST	**254.45**	**<10^−6^**	**16.703**	**<10^−6^**	1.41	0.22
vBNST	**177.10**	**<10^−6^**	**3.38**	**<0.005**	0.79	0.59
fuBNST	**128.96**	**<10^−6^**	**11.68**	**<10^−6^**	**2.44**	**<0.05**
pPVN	**298.44**	**<10^−6^**	**6.18**	**<0.0001**	**2.67**	**<0.02**
mPVN	**173.59**	**<10^−6^**	**5.61**	**<0.0001**	**2.48**	**<0.03**
cpEW	**85.11**	**<10^−6^**	**3.89**	**<0.005**	**3.36**	**<0.01**
DR	**157.87**	**<10^−6^**	**8.93**	**<10^−6^**	**8.03**	**<10^−5^**
S1	**956.36**	**<10^−6^**	**2.43**	**<0.04**	1.95	0.07

*Significant values are highlighted in bold. Abbreviations: medial (MeA), central (CeA), and BLA nuclei of the amygdala, oval (ovBNST), dorsolateral (dlBNST), dorsomedial (dmBNST), ventral (vBNST) and fusiform (fuBNST) divisions of the BNST, hypothalamic paraventricular nucleus, parvo- (pPVN) and magnocellular (mPVN) divisions, centrally projecting Edinger-Westphal nucleus (cpEW), dorsal raphe nucleus (DR), somatosensory barrel cortex area (S1). Results without significant interactions were further assessed by one-way ANOVAs for the control and stress group, respectively (see in Table 3)*.

**Table 3:** Summary of results obtained in one-way ANOVA on c-Fos cell counts.

Area	Condition	One-way ANOVA	
		Main effect of age	
		*F*	*p*
MeA	Control	***F*_(7,29)_ = 6.54**	***p* < 0.001**
	Stress	***F*_(7,28)_ = 28.70**	***p* < 10^−6^**
ovBNST	Control	***F*_(7,29)_ = 9.62**	***p* < 0.00001**
	Stress	***F*_(7,28)_ = 7.46**	***p* < 0.0001**
dlBNST	Control	***F*_(7,29)_ = 7.69**	***p* < 0.0001**
	Stress	***F*_(7,28)_ = 11.10**	***p* < 0.0001**
dmBNST	Control	***F*_(7,29)_ = 7.93**	***p* < 0.001**
	Stress	***F*_(7,28)_ = 11.17**	***p* < 0.0001**
vBNST	Control	*F*_(7,29)_ = 1.91	*p* = 0.10
	Stress	***F*_(7,28)_ = 3.06**	***p* < 0.02**
S1	Control	*F*_(7,29)_ = 1.58	*p* = 0.18
	Stress	***F*_(7,28)_ = 9.56**	***p* < 0.00001**

*Significant values are highlighted in bold. Abbreviations: medial nucleus of amygdala (MeA), oval (ovBNST), dorsolateral (dlBNST), dorsomedial (dmBNST), ventral (vBNST) divisions of the bed nucleus of stria terminalis (BNST), somatosensory barrel cortex area (S1)*.

**Table 4:** Summary of results obtained in Spearman’s rank correlation test on age and c-Fos cell counts.

Area	Condition	Spearman’s	
		*ρ*	*p*
MeA	Control	**−0.747**	**<0.001**
	Stress	**−0.828**	**<0.001**
CeA	Control	**−0.838**	**<0.001**
	Stress	**−0.575**	**<0.001**
BLA	Control	**−0.573**	**<0.002**
	Stress	0.075	0.662
ovBNST	Control	**−0.752**	**<0.001**
	Stress	**−0.696**	**<0.001**
dlBNST	Control	**−0.788**	**<0.001**
	Stress	**−0.707**	**<0.001**
dmBNST	Control	**−0.675**	**<0.001**
	Stress	**−0.777**	**<0.001**
vBSNT	Control	**−0.410**	**<0.05**
	Stress	−0.314	0.066
fuBNST	Control	**−0.609**	**<0.001**
	Stress	**−0.695**	**<0.001**
pPVN	Control	**−0.562**	**<0.001**
	Stress	**−0.508**	**<0.003**
mPVN	Control	**−0.480**	**<0.004**
	Stress	−0.208	0.224
cpEW	Control	**−0.430**	**<0.02**
	Stress	−0.199	0.244
DR	Control	−0.067	0.702
	Stress	−0.177	0.303
S1	Control	0.104	0.552
	Stress	−0.281	0.102

***ρ**: Spearman’s correlation coefficient; significant **ρ** and p values are highlighted in bold. Abbreviations: medial (MeA), central (CeA), and basolateral (BLA) nuclei of the amygdala, oval (ovBNST), dorsolateral (dlBNST), dorsomedial (dmBNST), ventral (vBNST) and fusiform (fuBNST) divisions of the bed nucleus of stria terminalis (BNST), hypothalamic paraventricular nucleus, parvo- (pPVN) and magnocellular (mPVN) divisions, centrally projecting Edinger-Westphal nucleus (cpEW), dorsal raphe nucleus (DR), somatosensory barrel cortex area (S1)*.

**Table 5:** Summary of c-Fos expression increase upon stress expressed as folds of changes for all examined age groups defined in months of age (M).

	1 M	1.5 M	2 M	3 M	6 M	12 M	18 M	24 M
MeA	**9.33**	**8.65**	**14.36**	**10.3**	**7.06**	**13.27**	**9.05**	**12.47**
*p*	6.7*10^−5^	1.8*10^−5^	3.6*10^−5^	3.1*10^−5^	3.4*10^−5^	2.2*10^−4^	4.0*10^−5^	3.4*10^−5^
CeA	1.66	2.21	**7.51**	**3.87**	**4.81**	**31.81**	**27.4**	**5.61**
p	0.921	0.248	1.5*10^−4^	1.4*10^−3^	2.1*10^−4^	1.5*10^−4^	1.5*10^−4^	2.21*10^−4^
BLA	3.06	**2.97**	**8.8**	**11.61**	**4.79**	**9.18**	**17.06**	**8.18**
*p*	0.193	**0.031**	1.5*10^−4^	**1.510^−4^**	4.3*10^−4^	1.7*10^−4^	1.5*10^−4^	1.6*10^−3^
ovBNST	1.36	1.7	**3.58**	1.85	**1.89**	**3.48**	**3.81**	2.45
*p*	*0.31*	*0.057*	3.5*10^−4^	*0.076*	**0.03**	***0.008***	***0.012***	*0.084*
dlBNST	**2.43**	**2.12**	**5.66**	2.13	**2.93**	**2.87**	**5.78**	**3.45**
*p*	***0.025***	***0.003***	2.0*10^−4^	*0.077*	***0.001***	***0.008***	6.1*10^−4^	***0.038***
dmBNST	**7.32**	**4**	**7.32**	**5.16**	**8.2**	**7.85**	**4.6**	**10.35**
*p*	***0.003***	9.5*10^−4^	7.0*10^−5^	3.5*10^−5^	5.7*10^−5^	8.7*10^−5^	4.6*10^−4^	***0.001***
vBNST	**5.05**	**4.5**	**6.85**	**2.94**	**5.54**	**5.25**	**8.25**	**4.18**
*p*	4.0*10^−4^	***0.005***	4.3*10^−4^	5.6*10^−4^	1.37*10^−5^	***0.011***	1.4*10^−4^	***0.003***
fuBNST	**3.62**	**3.77**	**5.99**	**3.25**	**2.51**	**4.4**	**6.61**	**4.88**
p	**0.011**	2.7*10^−3^	1.5*10^−4^	2.3*10^−3^	0.422	0.266	0.189	0.153
pPVN	**8.02**	**9.11**	**36.31**	**36.66**	**4.41**	**20.03**	**22**	**22.46**
p	**0.001**	6.2*10^−4^	1.5*10^−4^	1.5*10^−4^	**0.015**	2.6*10^−4^	1.5*10^−4^	1.5*10^−4^
mPVN	**3.73**	2.38	**10.78**	**6.61**	2.07	**6.32**	**9.17**	**7.05**
p	**0.041**	0.285	1.5*10^−4^	2.6*10^−4^	0.271	5.9*10^−3^	1.6*10^−4^	2.2*10^−4^
cpEW	1.028	2.2	**4.26**	**2.97**	2.33	**12.5**	3.36	2.23
p	1	0.1	1.6*10^−4^	2.8*10^−3^	0.79	4.9*10^−3^	0.32	0.286
DR	2.43	**3.51**	**10.77**	**7.19**	2.64	**20.67**	**7.1**	3.85
p	0.977	2.5*10^−4^	1.5*10^−4^	2.2*10^−3^	0.737	**0.027**	**0.016**	0.059
S1	**74.6**	**79.18**	**17.85**	**45.04**	**23.87**	**39.64**	**43.18**	**43.53**
*p*	5.1*10^−7^	4.8*10^−8^	***0.001***	8.9*10^−8^	8.3*10^−5^	1.3*10^−4^	2.2*10^−4^	2.3*10^−4^

*P values represent the results of the Tukey’s *post hoc* test. In six brain areas (MeA, ovBNST, dlBNST, dmBNST, vBNST, S1) two-way ANOVA found the main effect of stress significant, but did not reveal a significant interaction between age and stress (see also Table 2). Therefore, the stress induced c-Fos rise was assessed by t-test for each age groups. These p values were indicated in italics. Significant differences were highlighted by bold characters. Abbreviations: medial (MeA), central (CeA), and basolateral (BLA) nuclei of the amygdala, oval (ovBNST), dorsolateral (dlBNST), dorsomedial (dmBNST), ventral (vBNST) and fusiform (fuBNST) divisions of the bed nucleus of the stria terminalis (BNST), hypothalamic paraventricular nucleus, parvo- (pPVN) and magnocellular (mPVN) divisions, centrally projecting Edinger-Westphal nucleus (cpEW), dorsal raphe nucleus (DR), somatosensory barrel cortex area (S1)*.

To further support the link between age, CORT level and c-Fos immunoreactivity the statistical correlation between these variables was assessed by Spearman’s rank correlation test using SPSS 22 for Windows (SPSS Inc., Chicago, IL, USA). The statistical difference was considered significant if alpha was lower than 5%.

## Results

### Plasma Corticosterone Levels

To assess the HPA axis activity, the plasma CORT values were determined. Acute restrain stress exposure (*F*_(1,57)_ = 620.36; *p* < 10^−6^), age (*F*_(7,57)_ = 3.85; *p* < 0.005) and their interaction exerted significant effect (*F*_(7,57)_ = 3.57; *p* < 0.005) on CORT levels.

*Post hoc* tests revealed that stress exposure significantly elevated the CORT titer in all age groups (*p* < 0.005). The basal CORT levels were found to be constantly low till 6 months of age. In 12, 18 and 24 months old rats, the basal CORT level was doubled in comparison to the 3 months old controls (*p* < 0.05). This finding was supported by the Spearman’s rank correlation test (ρ = 0.427, *p* = 0.008). In stressed animals, the lowest serum hormone level was detected in the youngest, 1-month-old group. This value was significantly lower than that of other stress-exposed rats (*p* < 0.05), except the 3-month-old group (*p* = 0.09). From 1.5 month of age on the CORT value upon stress was nearly constant (ρ = 0.144, *p* = 0.413) throughout the lifespan (Figure 1).

### Neuronal Activity Patterns as Assessed by c-Fos Immunohistochemistry

To examine if a potential age-related neuronal activity pattern exists, eight age groups of rats were exposed to an acute restraint stress and 13 brain areas were examined by semi-quantitative c-Fos immunocytochemistry with the following results.

#### Nuclei of the Extended Amygdala

##### Medial Nucleus of the Amygdala

In the MeA, both stress (*F*_(1,57)_ = 517.11; *p* < 10^−6^) and age (*F*_(7,57)_ = 16.05 *p* < 10^−6^) exerted an influence on the number of c-Fos containing cells. As there was no significant interaction detectable (Table 2) between these factors, first we confirmed that stress elicited a significant c-Fos rise in all age groups by *t*-tests (Table 5, *p* < 0.001). Next, the control and stress cell counts were assessed separately. One-way ANOVA found the main effect of age in c-Fos cell counts significant in controls (*F*_(7,29)_ = 6.54, *p* < 0.001). We saw an age-related decline in the basal c-Fos values (Figure 2A), as in the youngest control group, we detected 15.62 ± 3.14 of c-Fos expressing cells with a decrease by the course of aging to 3.56 ± 0.84 cells in 24-month-old controls (Tukey’s *post hoc* test, *p* < 0.01). This has been validated by the rank correlation test also (ρ = −0.747; *p* < 10^−6^). Similarly, the main effect of age was significant in the stressed rats also (ANOVA: *F*_(7,28)_ = 28.70, *p* < 10^−6^). The magnitude of the c-Fos cell count reached its maximum at 2 months of age and then started to decline that became significant over 6 months of age (Tukey’s *post hoc* test, *p* < 0.04, Figure 2A). A strong negative correlation between age and stress-induced c-Fos cell counts was found (ρ = −0.828; *p* < 10^−6^). When comparing the magnitude of the c-Fos increase, we observed a 7–9-times elevation upon stress. A 14-times elevation was observed at 2 months of age. The relatively high, 13-times c-Fos elevation upon stress in old rats was related to the very low basal cell counts in these animals, as their c-Fos cell counts upon restraint was approximately only one fourth of those at 2 months of age.

##### Central Nucleus of Amygdala

The number of c-Fos expressing cells in the CeA was found to be altered by restraint stress (*F*_(1,57)_ = 234.27; *p* < 10^−6^) and age (*F*_(7,57)_ = 17.75; *p* < 10^−6^). ANOVA also found an age × stress interaction (*F*_(7,57)_ = 8.94; *p* < 10^−6^; Table 2).

The youngest control group exerted a 3.5-times higher number of c-Fos cells than 2 months old (*p* < 0.04) and older control rats (Figure 2B). By the course of aging, the basal c-Fos expression gradually decreased and almost disappeared in 12 and 18 months old animals. Restraint stress resulted in a 7.5-times elevation of c-Fos cell counts in 2-month-old rats (*p* < 10^−6^). The magnitude of the 3.5–5.6-times c-Fos elevation remained significant in the 3, 6 and 24-month-old rats (*p* < 0.0005). Due to the very low basal c-Fos expression in 12 and 18-month-old rats, the stress-induced rise appeared to be 31 (*p* < 10^−6^), and 27-fold (*p* < 10^−6^) respectively. Importantly, the 1.65 and 2.21-times elevation in the c-Fos expression in the 1 and 1.5-month-old rats did not reach the level of significance. The Spearman’s test revealed a correlation between age and basal c-Fos immunoreactivity (ρ = −0.838; *p* < 10^−6^) as well as age and post-stress (ρ = −0.575; *p* < 10^−6^) c-Fos cell counts.

##### Basolateral Nucleus of the Amygdala

According to the two-way ANOVA, both stress (*F*_(1,57)_ = 252.76; *p* < 10^−6^) and age (*F*_(7,57)_ = 3.45; *p* < 0.005) influenced the number of c-Fos positive cells in the BLA. A second order effect of age × stress interaction was also found to be significant (*F*_(7,57)_ = 3.02; *p* < 0.01; Table 2).

In the BLA, a relatively low (10.93 ± 2.57) number of c-Fos containing cells was found in 1-month-old control animals (Figure 2C). This basal c-Fos expression decreased with age also (ρ = −0.537; *p* = 0.001): except for the 6-month-old group, rats older than 1.5 month of age showed very low basal c-Fos immunoreactivity (i.e., 1.98 ± 0.36 cells per section, in 18-month-old rats). Due to this, the c-Fos rise in 12 and 18-month-old animals appeared to be 9.17 (*p* < 10^−6^) and 17.05 times (*p* < 10^−6^), respectively. Besides these, following restraint stress exposure, a 3–8-times elevation of c-Fos cell count was found in comparison to age matched controls (*p* < 0.01). The *post hoc* comparison of stressed groups revealed that the c-Fos reactivity of 2-month-old rats was higher than in the 1.5-month-old rats in the BLA (Figure 2C). In line with this, the Spearman’s test did not detect correlation between age and c-Fos cell counts upon restraint exposure (ρ = 0.075; *p* = 0.662).

##### The Oval Nucleus of the Bed Nucleus of the Stria Terminalis

In the ovBNST, according to two-way ANOVA the main effects of stress (*F*_(1,57)_ = 51.24; *p* < 10^−6^) and age (*F*_(7,57)_ = 13.61; *p* < 10^−6^) were significant, without interaction (Table 2).

The one-way ANOVA performed on the control c-Fos values found the main effect of age (*F*_(7,29)_= 9.62; *p* < 10^−6^) significant. There was a considerable (18.08 ± 4.93 cells) c-Fos expression in 1-month-old control rats, which gradually decreased by age and according to the *post hoc* tests appeared to be significant over the 6 months of age (Figure 3A). This correlation has been validated by the Spearman’s test also (ρ = −0.752; *p* < 10^−6^).

The highest restraint-induced c-Fos cell counts were observed in 1 and in 2-months-old rats. The 3.85-times rise in c-Fos expression caused by restraint appeared to be significant in 2-month-old animals (*p* < 0.02) only, meanwhile in the other groups this difference (1.35–3.8 times rise) did not reach the statistical power. Nevertheless, the one-way ANOVA (main effect of age *F*_(7,28)_ = 7.46; *p* < 0.0001) showed that age affects the c-Fos immunoreactivity here, and a negative correlation was found also (ρ = −0.696; *p* < 10^−6^).

##### The Dorsolateral Division of the Bed Nucleus of the Stria Terminalis

Two-way-ANOVA revealed that the number of c-Fos immunoreactive cells was affected by age (*F*_(7,57)_= 15.10; *p* < 10^−6^) and stress (*F*_(1,57)_ = 111.81; *p* < 10^−6^). The statistical value of stress × age interaction did not reach the significance (Table 2).

In control animals, one-way ANOVA found the main effect of age significant (*F*_(7,29)_ = 7.69; *p* < 0.00001). The 1-month-old control animals had 24.16 ± 6.11 c-Fos containing cells in the dlBNST. The 18-month-old (4.08 ± 0.94) and 24-month-old (3.45 ± 1.67) animals showed significantly lower c-Fos expression compared to the 1, 1.5 and 3-month-old control animals (*p* < 0.05), respectively (Figure 3B). The expected age-related decline appeared to be as a strong statistical correlation (ρ = −0.788; *p* < 10^−6^) according to the Spearman test.

The restraint exposure increased the c-Fos activity in all age groups significantly (*p* < 0.05). The highest number of c-Fos cells was observed in the 2 months old stressed animals. Age significantly influenced the magnitude of c-Fos expression in stressed rats (one-way ANOVA: *F*_(7,28)_ = 11.10; *p* < 0.000001). The Tukey’s *post hoc* test revealed that there was a significant decline in c-Fos reactivity from the 6 months of age on (*p* < 0.01, compared to 2 months of age). The rank correlation revealed that a negative correlation exists between age and c-Fos expression in stressed rats also (ρ = −0.707; *p* < 10^−6^).

##### Dorsomedial Division of the Bed Nucleus of the Stria Terminalis

The two-way ANOVA confirmed the main effects of stress (*F*_(1,57)_ = 254.452; *p* < 10^−6^) and age (*F*_(7,57)_ = 16.70; *p* < 10^−6^). As no interaction was detected (Table 2), we continued with one-way ANOVAs, and found the main effect of age significant on c-Fos signal both in control (*F*_(7,29)_= 7.93; *p* < 0.0001) and stressed (*F*_(7,28)_ = 11.17; *p* < 0.00001) groups. The correlation analysis revealed that old age was associated with lower c-Fos cell counts in both the control (ρ = −0.675; *p* < 10^−6^) and stress group (ρ = −0.777; *p* < 10^−6^).

The lowest basal c-Fos expression was detected in the 24-month-old group (*p* < 0.01, vs. 1-month control). Upon stress, the immunoreactivity of c-Fos was increased in all age groups (*p* < 0.001, Figure 3C). The c-Fos rise upon stress ranged between 4 to 6-fold, except for the oldest rats, where we found a 10-times elevation due to the very low basal c-Fos values. When the absolute cell counts were compared, 6-month-old and older rats displayed a significantly lower c-Fos response than 2-month-old rats (*p* < 0.001). In case of 6 (*p* = 0.098) and 18-month-old rats (*p* = 0.094), this difference did not reach the statistical significance.

##### The Ventral Nucleus of the Bed Nucleus of the Stria Terminalis

The main effects of stress (*F*_(1,57)_ = 177.10; *p* < 10^−6^) and age (*F*_(7,57)_ = 3.38; *p* < 0.005) on c-Fos cell count were found to be significant, without an interaction (Table 2).

The c-Fos cell count in the control groups did not depend on the age of rats (one-way ANOVA: *F*_(7,29)_ = 1.91; *p* = 0.10, Figure 3D), however the Spearman’s test found a weak negative correlation between age and basal c-Fos values (ρ = −0.401; *p* = 0.019). All groups upon restraint stress showed significantly higher c-Fos expression than their respective controls (*p* < 0.001). In stressed rats, one-way ANOVA found the main effect of age significant (*F*_(7,29)_ = 3.06; *p* < 0.016). The magnitude of the c-Fos rise upon stress was the greatest in the 2-month-old animals, while the smallest increase was detected in the oldest group, however, according to Tukey’s *post hoc* test, none of the stressed groups showed a statistical difference when compared to each other. In line with this, there was no significant correlation detectable between age and c-Fos expression in stressed rats in terms of c-Fos expression in the vBNST (ρ = −0.314; *p* = 0.066).

##### The Fusiform Division of the Bed Nucleus of the Stria Terminalis

In the fuBNST, the main effects of stress (*F*_(1,57)_ = 128.95; *p* < 10^−6^), age (*F*_(7,57)_ = 11.69; *p* < 10^−6^) and their interaction (*F*_(7,57)_ = 2.44; *p* < 0.05) were significant (Table 2).

The control groups showed low c-Fos immunoreactivity with the minimum at 18 months of age without statistical significance. Stress resulted in a three to four times rise of c-Fos immunoreactive cell numbers vs. controls in 1, 1.5 and 3-month-old rats (*p* < 0.05). The highest number of c-Fos immunoreactive cells was detected in the 2-month-old stressed rats corresponding to a six-time elevation compared to age-matched controls (*p* < 0.001). The age-related reduction in the magnitude of c-Fos expression from the 6 months of age on was significant. In this nucleus, the decline was that robust, that in 6, 12, 18 and 24-month-old animals the Tukey’s test did not find statistical differences anymore, despite the three to six-times difference between control and stress groups (Figure 3E). The test for statistical correlation confirmed an inverse relationship between c-Fos expression and age for both control (ρ = −0.609; *p* < 10^−6^) and stressed rats (ρ = −0.695; *p* < 10^−6^).

#### The Parvocellular Part of the Hypothalamic Paraventricular Nucleus

Two-way-ANOVA supported that the number of c-Fos positive neurons in the pPVN were altered both by restraint stress (*F*_(1,57)_ = 298.44; *p* < 10^−6^) and age (*F*_(7,57)_ = 6.18; *p* < 0.0001). In addition, the statistical analysis proved the significant effect of the two factors’ interaction (*F*_(7,57)_ = 2.67; *p* < 0.02, Table 2).

Based on *post hoc* tests, the basal c-Fos activity ranged between 2.72 ± 1.00 (24 months) and 25 ± 11.36 cells (6 months), which did not differ statistically (Figure 4A). Stress groups showed a significant, 4 to 36 times elevation of c-Fos cell counts compared to their respective controls (*p* < 0.05) throughout the lifespan. The highest increase we found in the 2 months old animals, with 36-times elevation compared to its control group (*p* < 10^−6^). The magnitude of the stress-induced PVN-c-Fos expression decreased significantly when 2 months old stressed rats were compared with 12 (*p* < 0.03), 18 (*p* < 0.05) or 24-month-old animals (*p* < 0.05).

The age-related decline of c-Fos immunoreactivity was confirmed by the Spearman’s rank correlation test both for control (ρ = −0.562; *p* < 10^−6^) and stressed (ρ = −0.508; *p* < 10^−6^) groups.

#### The Magnocellular Part of the Hypothalamic Paraventricular Nucleus

The c-Fos immunoreactive cell count was affected by stress (*F*_(1,57)_ = 173.59; *p* < 10^−6^) and age (*F*_(7,57)_ = 5.61; *p* < 0.0001) as well as by their interaction (*F*_(7,57)_ = 2.48; *p* < 0.03, Table 2) in this area also. The age-associated decline was also supported by the correlation analyses in control rats (ρ = −0.480; *p* = 0.003), while in stressed rats no significant correlation was detected (ρ = −0.208; *p* = 0.224).

Control rats showed basal cell counts between 0.96 ± 0.18 (18 months) and 6.28 ± 1.79 (6 months) cells. Stress evoked a significant six to nine-times rise in c-Fos cell counts in all ages, except for the 1.5 (*p* = 0.28) and 6-month-old groups (*p* = 0.27), where due to the slightly higher basal values the two-times elevation of c-Fos expression upon stress remained below the level of statistical power (Figure 4B). The highest c-Fos cell counts were detected in the 2-month-old groups, however, none of the other stress groups showed significantly lower cell counts (*p* > 0.05).

#### Centrally Projecting Edinger-Westphal Nucleus

The number c-Fos containing cells in the cpEW was influenced by stress (*F*_(1,57)_ = 85.12; *p* < 10^−6^), age (*F*_(7,57)_ = 3.89; *p* < 0.005) and their interaction (*F*_(7,57)_ = 3.36; *p* < 0.01; Table 2).

In 1-month-old controls, 19.50 ± 2.71 c-Fos cell count was detected (Figure 6G). It has to be pointed out, that this value was almost equal with the value (20.02 ± 5.49) found in the stress group (Figures 5A, *p* = 0.88). The lowest basal c-Fos cell count was detected in the 12-month-old rats (1.73 ± 0.48 cells), compared to 1-month-old controls (19.50 ± 2.71 cells; *p* < 0.02). Stress exposure caused a significant elevation of the c-Fos immunoreactivity in 2 (4.25-times rise), 3 (2.97-fold increase) and 12- (12.50-times elevation) month-old animals (Figure 5A). The magnitude of the age-related decline of c-Fos reactivity was less pronounced in this nucleus, as only the 6-month-old rats showed a significant reduction. This is mirrored by the results of our correlation tests also, which did not support an aging-related decline is stressed rats (ρ = −0.199; *p* = 0.244). In contrast, a weak, but significant (ρ = −0.430; *p* = 0.011) negative correlation was found between age and magnitude of c-Fos positive nuclei in the cpEW.

#### Dorsal Raphe Nucleus

The number c-Fos positive cells in DR was affected by the acute stress exposure (*F*_(1,57)_ = 157.87; *p* < 10^−6^) and age (*F*_(7,57)_ = 8.93; *p* < 10^−6^) as well as by their interaction (F_(7,57)_ = 7.69; *p* < 10^−5^, Table 2). Interestingly, when the rank correlation test was assessed, neither the control (ρ = −0.067; *p* = 0.702) nor the stressed animals (ρ = −0.177; *p* = 0.303) were found to show an ageing associated decline in the c-Fos expression.

Low c-Fos cell counts characterized the control groups with the maximum of 4.66 ± 1.59 cells at 1.5 months and with the minimum at 12 months of age (0.53 ± 0.2059 cells; Figure 5B). Restraint exposure led to significant, 4 to 20-fold elevation of c-Fos immunoreactive cells vs. age-matched controls (*p* < 0.05), except for the 1, 6 and 24-month-old animals. The restraint induced c-Fos immunoreactivity peaked in 2-month-old rats differing from all other stressed groups. No other age-related difference was found across the stressed groups.

#### The Somatosensory Barrel Cortex Area

To test if the age-related difference in c-Fos sensitivity was affected by a brain area that does not play a central role in stress adaptation response, the barrel cortex area layer IV was assessed also. This area was selected for evaluation as whiskers were bent by the conical end of the restrainer tubes when the rats were subjected to the restraint stress.

The assessment revealed that the c-Fos immunoreactivity was practically undetectable in layer IV in control rats. Here, we also observed that exposure to restraint caused a robust c-Fos response (two-way ANOVA: main effect of restraint *F*_(1,57)_ = 956.36, *p* < 10^−6^) and the main effect of age was also significant (*F*_(7,57)_ = 2.43, *p* < 0.03) without interaction. Testing the cell counts data of animals subjected to restraint by one-way ANOVA revealed the significant main effect of age (*F*_(7,28)_ = 9.56, *p* < 0.00004) also. *Post hoc* comparisons found that the youngest rats exerted approximately two-times higher cell counts (62.16 ± 2.71) than 6 (27.05 ± 3.57) and 12- (28.08 ± 3.17) month-old rats (Figure 6). In contrast to the other brain regions, our 18-month-old rats showed a slightly higher (35.08 ± 6.71) c-Fos response, compared to the 6 and 12-month-old animals, however this difference did not reach the statistical significance. In contrast, 24-month-old (58.04 ± 7.26) rats showed almost two-times higher c-Fos response than the 6 (*p* < 0.001) and 12- (*p* < 0.001) month-old counterparts which was very similar to the values counted in the youngest rats. This U-shaped dynamics of the c-Fos response with higher values in young age and senescence, but lower values in middle aged animals did not lead to a significant correlation between age and c-Fos expression (ρ = 0.281, *p* = 0.102).

### Correlation Analyses

As detailed above for the CORT values, the Spearman’s test found correlation between age and stressed CORT values. The correlation analysis data between age and c-Fos expression were addressed above for each brain area also. Taking the latter findings together, a negative correlation was found between age and c-Fos cell counts in control animals in 11 examined brain regions (MeA, CeA, BLA, ovBNST, dlBNST, dmBNST, vBNST, fuBNST, pPVN, mPVN, cpEW; see details in Table 4). The same comparison in stressed rats revealed that there is a negative correlation between c-Fos cell counts and age in seven brain areas (MeA, CeA, ovBNST, dlBNST, dmBNST, fuBNST and pPVN; see details in Table 4).

To further analyze our data, we also searched for correlations between CORT data and the c-Fos expression in the examined brain areas. The assessment of the basal CORT values and c-Fos revealed that these values correlate negatively only for most of the examined nuclei of the extended amygdala: CeA (ρ = −0.513; *p* = 0.002), MeA (ρ = −0.485; *p* = 0.003), ovBNST (ρ = −0.430; *p* = 0.01), dlBNST (ρ = −0467; *p* = 0.005), dmBNST (ρ = −0.388; *p* = 0.002), vBNST (ρ = −0.41; *p* = 0.01), fuBNST (ρ = −0.583; *p* < 10^−6^). In contrast, no significant correlation was found between stress-induced c-Fos cell counts and the CORT values in any examined brain areas.

## Discussion

In this work, we hypothesized that besides the HPA axis the c-Fos response of stress-related brain centers shows an age dependent dynamics. Using the acute restraint stress model, the c-Fos expression of 8 age groups of rats was evaluated. Our results support this hypothesis, as discussed below.

### The Validity of the Acute Restraint Stress Model

Acute restraint exposure is a reliable tool to test the effect of acute stress in rats (Coveñas et al., 1993; Kellogg et al., 1998; Gaszner et al., 2004, 2009; Viau et al., 2005; Sterrenburg et al., 2012) or mice (Marianno et al., 2017). Our protocol was successful as all studied stress-related centers reacted with a considerable elevation of c-Fos expression in 2-month-old animals, and except for the ovBSNT, in 3-month-old rats also. The stressor’s effectivity and the consequent HPA axis activation was proven by CORT RIA measurements as stress exposure increased glucocorticoid levels in all age groups. This is in line with earlier studies performed on young adult rats (Gaszner et al., 2004, 2009; Viau et al., 2005; Romeo et al., 2006).

### Stress- and Age-Related Dynamics of c-Fos Expression

#### Limitation of Data

First, although immunohistochemistry for c-Fos is a widely used tool to assess neuronal activation, it is known, that the expression of IEGs represents the results of a stimulus, which lies above a certain threshold (Worley et al., 1993) that leads to changes in gene expression and ultimately contributes to neuroplasticity (Kovács, 2008). However, the expression of IEGs is not associated with the general activity of nerve cells, otherwise one would see millions of c-Fos immunoreactive cells in all samples collected from naïve animals (Kovács, 2008). More specifically, it has been proven that neither the electrophysiological activity (Reisch et al., 2007) nor the higher metabolic rate of neurons (Duncan et al., 1993) are necessarily accompanied with c-Fos expression. On the other hand, we also know, that however for some cells a long-term tonic activity is characteristic, they do not express c-Fos (Dragunow and Faull, 1989; Hoffman et al., 1994) which is also a clear limitation of this, and similar studies.

Second, the activating protein 1 (AP-1) transcription factor mediates several types of responses, inducing both pathologic and physiologic stimuli (e.g., growth, migration, proliferation, regulation of stress signals, bacterial infections). Since AP-1 can be induced by various other factors (i.e., c-Jun, JUND, Atf4/5 (Alberini, 2009; Durchdewald et al., 2009; Vesely et al., 2009)), the functional significance of the c-Fos expression change cannot be identified by this method (Kovács, 1998).

Third, the technique may not visualize potentially highly important inhibitory changes (Bowers et al., 1998; Choi et al., 2007). Nevertheless, the acute restraint stress-induced c-Fos expression mapping in line with previous studies (Dayas et al., 2001; Crane et al., 2005; Sterrenburg et al., 2012) is a powerful technique to assess the activation of stress associated centers. Therefore, with respect to all limitations our data provide valuable insights into the age- and stress-related activity of stress-related centers.

### Age-Related Pattern of Stress-Induced c-Fos Expression

All of the studied nuclei were significantly activated by restraint stress at 2 months of age as exemplified by higher expression level of c-Fos (see Table 5) in line with earlier works (Briski and Gillen, 2001; Dayas et al., 2001; Crane et al., 2005; Sterrenburg et al., 2012).

Although the magnitude of c-Fos expression did not differ significantly when comparing stressed young rats (i.e., 1, 1.5, 2 and 3 months of age), except for the DR nucleus, the highest c-Fos cell count was found in the 2-month-old animals. This may be explained by the increased stress sensitivity period of the brain, which is characteristic for late adolescence/post pubertal period (Kellogg et al., 1998; McCormick et al., 2010). This age-altered sensitivity difference in some nuclei may refer to vulnerable periods of particular brain areas. The age-dependent susceptibility of stress-centers might explain the altered effectivity of potent stressors, which may result in psychiatric disorders (McCormick et al., 2010).

The c-Fos response to restraint stress decreased with age gradually. When compared with the 2 months old stress group, the *post hoc* test found this decline significant in nine nuclei (i.e., MeA, CeA, ovBNST, dlBNST, dmBNST, fuBNST, cpEW and DR), however, for two areas (i.e., BLA and vBNST) this *post hoc* comparison revealed that the difference remained below the level of significance. The comparison revealed that the ageing-related reduction in the magnitude of c-Fos expression is significant over 3 months of age in the CeA and DR, over the 6 months of age for the MeA, ovBNST, dmBNST, fuBNST, PVN and for the cpEW only for the 6 months old group. In the case of the dlBNST, the reduction was significant over 12 months of age.

If there is a true correlation between age and c-Fos, the results were further analyzed by Spearman’s test. The dynamics of control and post-stress c-Fos values were assessed separately. This statistical tool supported a negative correlation between age and c-Fos expression in eleven brain regions, while the correlation upon stress was found in seven regions only.

One may argue here, that only the relatively high maximal c-Fos values at 2 months of age may cause the age dependency found in this experiment. In order to test this possibility, the results have been re-assessed by excluding this group, and even after this action, ANOVA would confirm the main effect of age in all brain regions. The correlation analyses after omitting the 2 months old group would reveal that the c-Fos response declines with age in the same seven areas (MeA, CeA, ovBNST, dlBNST, dmBSNT, fuBNST, pPVN).

Comparing the magnitude of the c-Fos expression in stressed animals at 2 months age in the studied nuclei, the highest expression-rise was detected in the pPVN. This approximately 36-fold elevation is not surprising since the pPVN is the key regulator of HPA axis (Petrov et al., 1994; Pacak et al., 1995; Romeo et al., 2006). As the studied limbic centers provide dense afferent connections converging to the PVN (Pacak et al., 1995; Carrasco and Van de Kar, 2003), their increased c-Fos expression in 2-month-old rats may refer to their regulatory contribution to the strong activation of PVN resulting in c-Fos rise here also.

Correlation analyses between post stress c-Fos and CORT values in the course of aging did not reveal strong associations in any examined brain regions. This suggests that the c-Fos response is not a reliable indicator of the HPA axis activity. As stated above, the c-Fos labeling for assessing the neuronal activity has its limitations, which may explain these discordant results. On the other hand, the HPA axis activity is influenced by numerous other, in this study not examined factors. For instance, glucocorticoid response, glucocorticoid and mineralocorticoid receptor density (for review see de Kloet et al., 2016), and other brain areas (hippocampus, thalamus, cortex; for review see Ulrich-Lai and Herman, 2009) might have influenced the HPA axis activity which finally resulted in a stable CORT response from 2 months of age till senescence. Based on these we propose that the age- and brain area specific dynamics of stress recruited brain areas is required for the normal stress adaptation response. The age-related alteration in the response to stress might contribute to the dysregulation of the HPA-axis, which ultimately leads to mood disorders.

The comparison of the c-Fos rise by two-way ANOVA found significant age × stress interactions in seven brain areas (BLA, CeA, fuBNST, pPVN, mPVN, cpEW, DR). When we analyzed the magnitude difference in c-Fos expression across age groups for each area, we saw that the difference between the smallest and greatest rise was 5 to 19 times in these regions. Correlation analyses supported that upon stress out of these areas a negative correlation between age and c-Fos response was found in the CeA, fuBNST and pPVN. This suggests that the age-related change in the stress sensitivity of the pPVN might be underlined by the CeA and fuBNST. In line with this, the CeA was shown to be required for the glucocorticoid response to stress, their lesioning leads to decreased HPA axis activity (Choi et al., 2007) and both nuclei were shown to possess dense connectivity with the PVN (Dong et al., 2001).

In contrast, in six regions (S1, MeA, ovBNST, dlBNST, dmBNST, vBNST) no interaction was found. We compared the magnitude of the c-Fos rise in these areas across all age groups, and saw that the difference in the magnitude of the c-Fos rise across all age groups was only 2–3 times. Although the one-way ANOVAs supported that age influences the c-Fos expression in these areas upon stress as well, the magnitude of the c-Fos rise was more stable in the latter areas and the correlation test did not find an age-related decline in S1, vBNST. Based on these we propose that the MeA, ovBNST, dlBNST, dmBNST and vBNST contribute to a lesser extent to age related differences, on the other hand they may contribute to stabilization of the HPA axis response.

The primary somatosensory barrel cortex (S1) region was selected as an area that is not specifically involved in the stress adaptation response. The goal was here to see whether the age-related dynamics of the c-Fos response observed in the stress-recruited areas differs from that in the barrel cortex. In line with earlier studies at mRNA level (Girotti et al., 2006), restraint stress induced the expression of c-Fos protein in this area in the current experiment. Similarly, the effect of whisker stimulation was shown to induce strong c-Fos expression in the lamina IV of the S1 (Bisler et al., 2002; Lecrux et al., 2011). Our results show that age does affect the c-Fos response here also, however, no negative correlation was found here, as a U-shaped dynamics in age is characteristic for this brain region (Figure 6A). It has to be stated that in this experimental setup we do not know how far does the psychological/emotional effect of stress influence c-Fos in S1 and to what extent do the mechanical stimuli on the whiskers contribute to the c-Fos expression. Nevertheless, our finding suggests that the age-related decline in the above described brain regions may not be explained by a simple aging-related decline in the sensitivity of the sensory systems. Another argument to support this would be that the largest decline in the c-Fos response occurs in rats of middle age (6 and 12 months), and the magnitude of the further decline in senescence (18 and 24 months) is relatively low. Therefore, we propose that the observed age-related dynamics is characteristic for the stress-recruited brain areas, but not for all brain regions and they are not related to the loss of sensory sensitivity in old age.

### Comparison of Basal c-Fos Expression

An unexpected finding of this study was that the c-Fos expressions of control groups were found to be a function of age as well. The c-Fos is continuously expressed at low levels in the central nervous system (Fevurly and Spencer, 2004). It is known for some areas that there is a well-detectable basal c-Fos expression, such as in the suprachiasmatic nucleus, PVN, hippocampus, amygdala and preoptic area (Kellogg et al., 1998; Meyza et al., 2007; Kovács, 2008). When comparing the examined 13 areas, 10 (i.e., dlBNST (Figure 3B), dmBNST (Figure 3C), vBNST (Figure 3D), fuBNST (Figure 3E), MeA (Figure 2A), BLA (Figure 2C), pPVN, mPVN (Figures 4A, 7E,F), DR (Figures 5B, 6I,J), S1 (Figure 6A)) showed very low basal c-Fos expression compared to the stressed groups throughout lifespan. In contrast, three nuclei (CeA (Figures 2B, 7A,B), ovBNST (Figures 3A, 7C,D), cpEW (Figures 4A, 7G,H)) exert higher basal expression in the 4 weeks old group, and the values from the 6th week of age on showed similar basal c-Fos expression pattern as that of other examined regions. The reason for this relatively high basal c-Fos expression is unknown. Since these three areas express stress-recruited neuropeptides (i.e., CRF in the ovBNST and CeA moreover urocortin1 (Ucn1) in the cpEW), one could speculate that the post-weaning period in rats requires increased basal c-Fos expression activity in these stress centers to help the adaptation response in the beginning of the vulnerable juvenile period (Horovitz et al., 2012). If this relatively high basal c-Fos expression is indeed characteristic for the CRF and/or Ucn1 neurons requires further confirmation by co-localization studies.

Regarding the considerable basal c-Fos expression, one may argue that the higher c-Fos cell counts could be caused by an unwanted stress exposure of control animals. However, two observations of the current study are against this expectation. The highly stress-sensitive pPVN does not show elevated basal c-Fos values in the same control animals; on the other hand, the rats’ low basal CORT levels also mirror low HPA axis activity.

### Conclusion and Future Perspective

To our knowledge, this study is the first to provide a systematic comparison of both basal and acute restraint exposure-induced c-Fos expression in the main stress-related brain areas of Wistar rats in eight age groups from young age till senescence. Since the c-Fos expression was found to be a function of age both in control and stressed groups, the main methodological message of this study is that the outcomes of similar experimental procedures are highly age and brain region sensitive. Since the magnitude of the basal c-Fos expression was found to be brain area and age specific, one has to consider that young animals may not be reliable controls in studies performed on specific brain regions (i.e., CeA, BSTov, cpEW). A more precise morphological characterization and the determination of functional significance of high basal c-Fos expression in young rats awaits further experimentation.

Our results also provide evidence that the acute stress responsibility of seven (MeA, CeA, ovBNST, dlBNST, dmBNST, fuBNST and pPVN) examined brain areas negatively correlates with age. Therefore, their contribution to the control of the HPA axis and that of other stress-related systems may also be a function of age. Further extensive systematic research is required to test the age-dependent contribution of these areas to the age dependency of stress adaptation response. Similar studies involving also chronic stress models may help to understand why stress-related mood disorders develop in vulnerable periods of life more frequently.

## Author Contributions

LK evaluated the results, performed statistics, prepared the figures and wrote the draft manuscript. JS and AN performed the animal experiments, immunolabeling, digital imaging and cell counting. VC performed the CORT measurement. BG designed the experiments, collected the blood samples, performed perfusion, supervised the tissue preparation, imaging, selected the images containing the areas of interest, helped with figure preparation and supervised the manuscript.

## Conflict of Interest Statement

The authors declare that the research was conducted in the absence of any commercial or financial relationships that could be construed as a potential conflict of interest.

## Abbreviations

BNST: bed nucleus of the stria terminalis
BLA: basolateral nucleus of amygdala
CeA: central nucleus of amygdala
CRF: corticotropin-releasing factor
cpEW: centrally projecting Edinger-Westphal nucleus
CORT: corticosterone
DAB: 3,3’diamino-benzidine
dlBNST: dorsolateral division of the bed nucleus of the stria terminalis
dmBNST: dorsomedial division of the bed nucleus of the stria terminalis
DR: dorsal raphe nucleus
fuBNST: fusiform division of the bed nucleus of the stria terminalis
HPA: hypothalamus-pituitary-adrenal axis
ANOVA: analysis of variance
MeA: medial nucleus of amygdala
mPVN: magnocellular division of the paraventricular nucleus of the hypothalamus
NGS: normal goat serum
ovBNST: oval division of the bed nucleus of the stria terminalis
PBS: phosphate buffered saline
pPVN: parvocellular division of the paraventricular nucleus of the hypothalamus
RIA: radioimmunoassay
S1: somatosensory barrel cortex area
Ucn1: urocortin 1
vBNST: ventral division of the bed nucleus of the stria terminalis.
